# The association between maternal HbA1c and adverse outcomes in gestational diabetes

**DOI:** 10.3389/fendo.2023.1105899

**Published:** 2023-03-16

**Authors:** Marie Parfaite Uwimana Muhuza, Lixia Zhang, Qi Wu, Lu Qi, Danqing Chen, Zhaoxia Liang

**Affiliations:** ^1^ Obstetrical Department, Women’s Hospital, School of Medicine, Zhejiang University, Hangzhou, China; ^2^ Department of Epidemiology, School of Public Health and Tropical Medicine, Tulane University, New Orleans, LA, United States

**Keywords:** gestational diabetes mellitus, obesity, gestational weight gain, pre-pregnancy body mass index, glycated hemoglobin A1c, adverse outcomes

## Abstract

**Background:**

The role of HbA1c in women with gestational diabetes mellitus (GDM) is still unclear, particularly in the Asian population.

**Aim:**

To investigate the association between HbA1c levels and adverse outcomes considering maternal age, pre-pregnancy body mass index (BMI), and gestational weight gain (GWG) in women with GDM.

**Method:**

A retrospective study included 2048 women with GDM and singleton live births. Using logistic regression, the associations between HbA1c and adverse pregnancy outcomes were assessed.

**Result:**

Compared to women with HbA1c ≤ 5.0%, HbA1c was significantly associated with macrosomia (aOR 2.63,95%CI1.61,4.31), pregnancy-induced hypertension (PIH, aOR 2.56,95%CI1.57,4.19), preterm birth (aOR 1.64,95%CI 1.05,2.55), and primary Cesarean section (primary C-section, aOR1.49,95%CI1.09,2.03) in GDM women with HbA1c ≥5.5% while significantly associated with PIH (aOR 1.91,95%CI1.24,2.94) in women with HbA1c 5.1-5.4%. The associations between HbA1c and adverse outcomes varied with maternal age, pre-pregnancy BMI, and GWG. In women aged ≤29 years, there’s significant association between HbA1c and primary C-section when HbA1c was 5.1-5.4% and ≥5.5%. In women aged 29-34 years and HbA1c ≥5.5%, HbA1c was significantly associated with macrosomia. In women aged ≥35 years, there’s significant association between HbA1c and preterm birth when HbA1c was 5.1-5.4% and macrosomia and PIH when HbA1c ≥5.5%. In pre-pregnant normal-weight women, HbA1c was significantly associated with macrosomia, preterm birth, primary C-section, and PIH when HbA1c ≥5.5% while HbA1c was significantly associated with PIH when HbA1c was 5.1-5.4% . In pre-pregnant underweight women with HbA1c 5.1-5.4%, HbA1c was significantly associated with primary C-section. HbA1c was significantly associated with macrosomia among women with inadequate GWG or excess GWG and HbA1c≥5.5%. In women with adequate GWG, there’s significant association between HbA1c and PIH when HbA1c was 5.1-5.4% and ≥5.5% .

**Conclusion:**

Conclusively, HbA1c at the time of diagnosis is significantly associated with macrosomia, preterm birth, PIH, and primary C-section in Chinese women with GDM.

## 1 Introduction

Gestational diabetes mellitus (GDM) is carbohydrate intolerance resulting in hyperglycemia during pregnancy without prior history of diabetes (Type 1 or Type 2) (1). It is screened using fasting plasma glucose (FPG), 1-h postprandial glucose (PG), 2-h PG of 75g oral glucose tolerance test (OGTT) during 24-28 weeks, according to the International Association of Diabetes and Pregnancy Study Groups (IADPSG) criteria (2). The availability of screening for gestational diabetes in the past years has increased the detection rate of GDM (3). The incidence of GDM in China is 14.8%, caused by increasing weight gain, maternal age, family history, and many other factors linked with the pregnancy period of women (4). The increase in gestational diabetes incidence and its association with Type 2 diabetes remains crucial (5). GDM is associated with both short and long-term pregnancy adverse outcomes, including macrosomia, large for gestational age (LGA), preeclampsia, primary Cesarean section (C-section), shoulder dystocia, preterm birth, postpartum diabetes mellitus and risk of Type 2 diabetes in offspring (6–8).

HbA1c is used in diagnosing, treatment, preventing, and detecting progress of diabetes (9). In women with hyperglycemia, glycated hemoglobin A1c (HbA1c) level has been associated with birthweight, primary C-section, hypoglycemia, cord-serum C-peptide, pre-eclampsia, preterm birth, the sum of skin folds, percent body fat >90^th^ percentile (10). It has been reported that adverse outcomes in early pregnancy can be predicted by HbA1c (11–13) as well as in GDM pregnant women (14, 15). But different HbA1c cut-offs have been used in past studies to predict adverse outcomes in GDM pregnancy. HbA1c level ≥5.0% was used to predict neonatal complications and ≥6.2% to predict postpartum diabetes mellitus (14, 16). HbA1c might be useful in predicting adverse outcomes in GDM and studies indicating the association between HbA1c and adverse outcomes have been conducted in Caucasian women with GDM (17). However, there is a lack of enough evidence in the Asian population.

This retrospective study aims to investigate the relationship between HbA1c levels and adverse pregnancy outcomes considering maternal age, pre-pregnancy body mass index (BMI), and gestational weight gain (GWG) among GDM women, which might provide evidence for the prevention of adverse outcomes in GDM pregnant women.

## 2 Methods

### 2.1 Study design and population

A retrospective study was conducted among women with gestational diabetes who received regular prenatal care and delivered at the Women’s Hospital, School of Medicine, Zhejiang University from 1-July-2017 to 30-June-2018. Women who were diagnosed with GDM by OGTT in the second trimester of pregnancy, delivered a live singleton more than 28 gestational weeks, and had complete medical records were included. Women who had a prior history of diabetes mellitus, chronic diseases (hypertension, liver, kidney, heart, lung, and other major organ diseases), autoimmune diseases (Sjogren’s syndrome, anticardiolipin syndrome, myasthenia gravis), or tumors were excluded. Finally, 2048 GDM women were included in this study.

Relevant information about pregnant women, including age, height, weight before pregnancy (within one month before pregnancy), weight gain during pregnancy, gravidity, parity, OGTT value (FPG, 1-h PG, 2-h PG), HbA1c, mode of delivery, gestational week of delivery, neonatal birth weight, pregnancy complications such as macrosomia, pregnancy-induced hypertension (PIH, including gestational hypertension, preeclampsia, eclampsia) was obtained.

### 2.2 Diagnostic criteria

#### 2.2.1 GDM diagnostic criteria

GDM was diagnosed according to IADPSG criteria by 75g OGTT in the second trimester of pregnancy by measurement of FPG, 1-h PG, and 2-h PG. OGTT and HbA1c tests were performed in the morning after overnight fasting of at least 8 hours at 24-28 weeks of gestation. G lucose level was measured using a clinical chemistry system (Beckman Coulter AU5800) automatic analyzer. HbA1c was measured by high-performance liquid chromatography (HPLC) on an automated glycosylated hemoglobin analyzer (HLC-723G8), which has been certified by the National Glycohemoglobin Standardization Program (NGSP) to conform to the results of the Diabetes Complications and Control Trial and standardized according to International Federation of Clinical Chemistry (IFCC) reference system.

#### 2.2.2 BMI

BMI was calculated as pre-pregnancy weight in kilograms(kg) divided by the square of height in meters(m). Pre-pregnancy BMI was categorized into underweight (<18.5 kg/m^2^), normal weight (18.5 kg/m^2^-23.9 kg/m^2^), overweight (24.0 kg/m^2^-27.9 kg/m^2^), and obese (≥28.0 kg/m^2^) groups according to Chinese criteria. (National Health Commission of the People’s Republic of China: Criteria of Weight for Adults. [(accessed on 10 August 2021)];2013 Available online: http://www.nhc.gov.cn/ewebeditor/uploadfile/2013/08/20130808135715967).

#### 2.2.3 GWG

GWG was the difference between pre-delivery and pre-pregnancy weight. According to the standard definition of the Institute of Medicine (IOM) guidelines in 2009 (18), appropriate GWG was 12.5-18.0 kg for underweight, 11.5-16.0 kg for normal weight, 7.0-11.5 kg for overweight and 5.0-9.0 kg for obesity respectively. Additionally, falling below the thresholds was defined as inadequate GWG, while exceeding the thresholds was defined as excessive GWG.

#### 2.2.4 Adverse pregnancy outcomes

Neonates were defined as LGA if their birth weight was >90th percentile based on national population references for age and sex. Neonates with gestational age ≥ 28 weeks and < 37 weeks were considered as preterm neonates. Neonates with birth weight ≥4000g were defined as macrosomia. PIH was diagnosed in women with no previous history of hypertension with systolic blood pressure (SBP) ≥140 mmHg and diastolic blood pressure (DBP) ≥90 mmHg on two occasions at least 4 hours apart after 20 gestational weeks with or without proteinuria (19).

### 2.3 Statistical analysis

Maternal and neonatal demographic and clinical features were reported as frequency (%) or means ( ± SD). Categorical variables, including maternal age groups, parity, gravidity, pre-pregnancy BMI group, GWG groups, and difference in the incidence of adverse pregnancy outcomes among HbA1c groups, were evaluated by chi-squared test. Continuous data, including birthweight, FPG, 1h-PG, 2h-PG, and maternal age, were evaluated using one-way ANOVA. HbA1c level was divided into three different categories by quartiles, which included ≤25th (5.0%, 31mmol/mol), 25th-75th (5.1-5.4%, 32-36mmol/mol) and ≥75th (5.5%, 37mmol/mol). Logistic regression was used to explore the association between HbA1c level and adverse outcomes in different maternal age groups, pre-pregnancy BMI groups, and GWG groups. Two-sided *p-values* less than 0.05 were considered significant. All statistical analyses were done with SPSS 26.0 software.

## 3 Results

### 3.1 General clinical characteristics and pregnancy outcomes of three HAb1c groups

**Figure 1:**
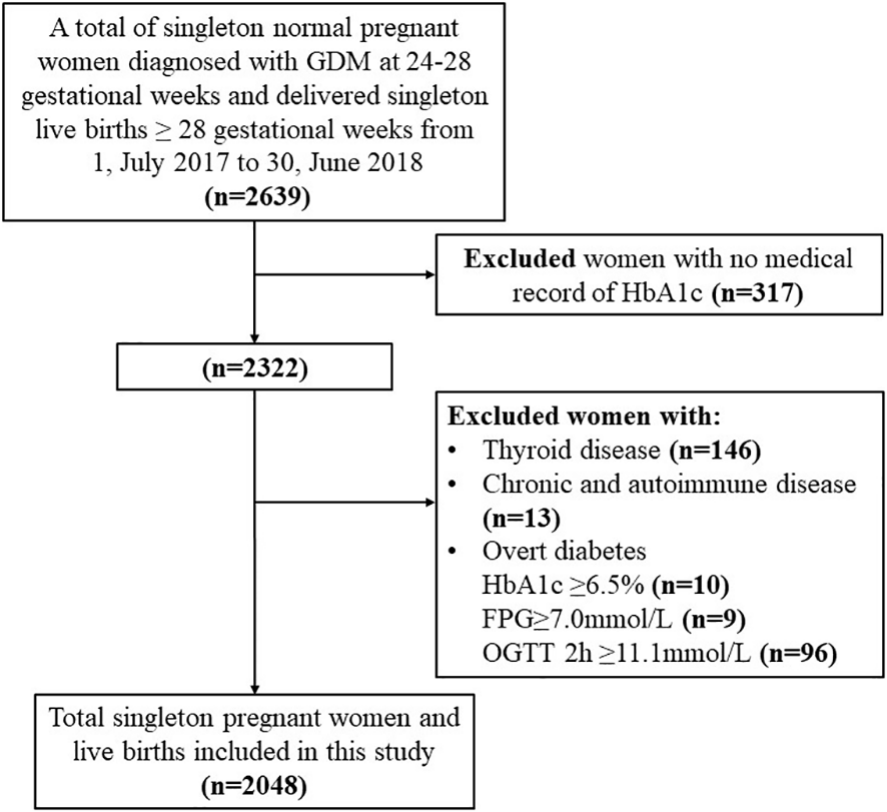
Flow chart of the study population. Demonstrates the inclusion and exclusion criteria of our study population; glycated hemoglobin A1c (HbA1c); gestational diabetes mellitus (GDM); fasting plasma glucose (FPG); 2hPG (2-hour plasma glucose); Oral glucose tolerance test (OGTT); chronic diseases (hypertension, liver, kidney, heart, lung and other major organ diseases, or tumors); autoimmune diseases (Sjogren's syndrome, anticardiolipin syndrome, myasthenia gravis).

Our study enrolled 2048 women with GDM of live singleton births without missing data (**Figure 1**). There were significant differences in maternal age (p<0.001), pre-pregnancy BMI (p<0.001), GWG (p<0.001), parity (p=0.001), and gravidity (p=0.001) among three HbA1c groups (**Table 1**). There were also significant differences in the incidence of macrosomia (p<0.001), preterm birth (p=0.020), primary C-section (p<0.007), and PIH (p<0.001) among HbA1c groups. Additionally, higher incidences of adverse outcomes (macrosomia, preterm birth, primary C-section, and PIH) were observed in GDM women with HbA1c ≥5.5% at the time of GDM diagnosis compared to other HbA1c groups. There was no significant difference in the incidence of LGA among HbA1c groups (**Table 1**).

**Table 1:** Obstetrical characteristics by HbA1c groups in GDM^1^.

	HbA1c% (mmol/mol)			
Characteristic	≤5.0 (31mmol/mol) (n=755)	5.1≤HbA1c≤ 5.4 (32– 36 mmol/mol) (n=942)	≥5.5 (37 mmol/mol) (n=351)	p^2^
**Birth weight**	3248.2 ± 451.0	3291.9± 491.1	3403.0 ± 593.5	<0.001
**Maternal Age**	31.7 ± 4.2	32.6 ± 4.4	33.7 ± 4.8	<0.001
**<29**	35.4%	28.0%	19.7%	
**30-34**	37.5%	38.0%	36.2%	
**≥35**	27.2%	34.0%	44.2%	
**Gravidity**				0.001
**0**	34.7%	29.1%	26.5%	
**1 to 2**	53.2%	54.1%	52.7%	
**≥3**	12.1%	16.8%	20.8%	
**Parity**				0.001
**Nullipara**	418 (55.4%)	437(46.4%)	155 (44.2%)	
**Multipara**	337(44.6%)	505(53.6%)	196 (55.8%)	
**Pre-pregnancy BMI**				<0.001
**Normal**	70.7%	68.9%	53.6%	
**underweight**	18.5%	10.0%	4.6%	
**Overweight**	9.5%	17.7%	30.5%	
**Obese**	1.2%	3.4%	11.4%	
**OGTT**				
**FPG**	4.5 ± 0.4	4.7 ± 0.5	5.0± 0.6	<0.001
**1h-PG**	9.8± 1.3	10.0 ± 1.1	10.3 ± 1.4	<0.001
**2h-PG**	8.7 ± 1.1	8.7 ± 1.2	8.8 ± 1.2	0.031
**GWG**				<0.001
**Adequate**	40.9%	43.4%	35.0%	
**Inadequate**	43.4%	35.5%	34.5%	
**Excess**	15.6%	21.1%	30.5%	
**Macrosomia**	4.4%	6.2%	13.4%	<0.001
**Preterm birth**	7.4%	10.0%	12.5%	0.020
**Primary C-section**	24.0%	27.2%	33.0%	0.007
**PIH**	4.2%	8.8%	15.1%	<0.001
**LGA**	0.9%	1.1%	1.4%	0.756

**^1^** BMI in kg/m^2;^ values were expressed as mean ± standard deviation (SD) or n (%) unless indicated otherwise. Glycated hemoglobin A1c, HbA1c; gestational diabetes mellitus, GDM; oral glucose tolerance test, OGTT; fasting plasma glucose, FPG; postprandial glucose, PPG; pregnancy induced hypertension, PIH; gestational weight gain, GWG.

**^2^** Based on chi-square test.

### 3.2 Association between HbA1c and adverse outcomes

In GDM women with HbA1c ≥5.5%, HbA1c was significantly associated with preterm birth (aOR 1.64,95%CI1.05,2.55), macrosomia (aOR 2.63,95%CI1.61,4.31), and primary C-section (aOR 1.49,1.09,2.03) compared to their counterparts with HbA1c ≤5.0%. Interestingly, both GDM women with HbA1c 5.1%-5.4% and HbA1c ≥5.5% had significantly increased risk of PIH (aOR 1.91, 95%CI 1.24,2.94; aOR 2.56, 95%CI 1.57,4.19), respectively compared to their counterparts with HbA1c ≤5.0% (**Table 2**).

**Table 2:** Association between HbA1c and adverse outcomes.

	HbA1c% (mmol/mol)		
Adverse outcomes	≤5.0 (31mmol/mol) (n=755)	5.1≤HbA1c≤ 5.4 (32–36mmol/mol) (n=942)	≥5.5 (37mmol/mol) (n=351)
	aOR (95% CI)	aOR (95% CI)	aOR (95% CI)
Preterm birth (n=194)	Ref	1.39 (0.97,1.97)	1.64 (1.05,2.55)*
Macrosomia (n=138)	Ref	1.26 (0.80,1.97)	2.63 (1.61,4.31)*
PIH (n=168)	Ref	1.91 (1.24,2.94)*	2.56 (1.57,4.19)*
Primary C-section (n= 553)	Ref	1.23 (0.97,1.56)	1.49 (1.09,2.03)*

**^1^** Glycated hemoglobin A1c (HbA1c) adjusted odds ratio (aOR), confidence interval (CI), pregnancy induced hypertension (PIH), and Ref represents the reference.

**^2^** Multiple logistic regression model was adopted and adjusted for gravidity, parity, maternal age, gestational weight gain (GWG), and pre-pregnancy BMI. *p < 0.05.

### 3.3 Association between HbA1c and adverse outcomes in different maternal age groups

There w ere significantly positive associations between HbA1c level and primary C-section in women aged ≤29 years with HbA1c 5.1 - 5.4% (aOR 1.51,95%CI1.00,2.29) or HbA1c ≥5.5% (aOR 2.35, 95%CI 1.22,4.53) compared to their counterparts with HbA1c ≤5.0%. Interestingly, young women aged ≤29 years showed an increased risk of PIH when their HbA1c was≥5.5% (aOR 3.53,95%CI1.34,9.30). Additionally, women aged ≥35 years with HbA1c ≥5.5% also showed an increased risk of PIH (aOR 2.56,95%CI1.13,5.78) compared to women ≥35 years with HbA1c ≤5.0%. HbA1c ≥5.5% was significantly associated with macrosomia among women aged 30 -34 years old (aOR2.48,95%CI1.16,5.31) and those aged ≥35 years (aOR 5.52, 95%CI 2.00,15.24) compared to HbA1c ≤5.0% (**Table 3**).

**Table 3:** Association between HbA1c and adverse outcomes in different maternal age groups.

	HbA1c% (mmol/mol)		
Maternal age	≤5.0 (31mmol/mol) (n=755)	5.1≤HbA1c≤ 5.4 (32–36mmol/mol) (n=942)	≥5.5 (37mmol/mol) (n=351)
	aOR (95% CI)	aOR (95% CI)	aOR (95% CI)
≤29 years (n=600)			
Preterm birth	Ref	1.02 (0.52,2.02)	2.26 (0.93,5.45)
Macrosomia	Ref	1.32 (0.62,2.78)	1.12 (0.38,3.31)
PIH	Ref	2.09 (0.95,4.60)	3.53 (1.34,9.30)*
Primary C-section	Ref	1.51 (1.00,2.29)*	2.35 (1.22,4.53)*
30-34 years (n=768)			
Preterm birth	Ref	1.07 (0.59,1.94)	1.48 (0.71,3.07)
Macrosomia	Ref	0.87 (0.42,1.78)	2.48 (1.16,5.31)*
PIH	Ref	1.89 (0.91,3.91)	2.04 (0.88,4.69)
Primary C-section	Ref	0.98 (0.67,1.44)	1.18 (0.72,1.96)
≥ 35 years (n=680)			
Preterm birth	Ref	2.11 (1.14,3.90)*	1.43 (0.67,3.03)
Macrosomia	Ref	2.46 (0.89,6.79)	5.52 (2.00,15.24)*
PIH	Ref	1.73 (0.81,3.69)	2.56 (1.13,5.78)*
Primary C-section	Ref	1.23 (0.78,1.94)	1.44 (0.85,2.43)

**^1^** Glycated hemoglobin A1c, HbA1c; adjusted odds ratio, aOR; confidence interval, CI; pregnancy induced hypertension, PIH; reference, Ref.

**^2^** Multiple logistic regression model was adopted and adjusted for gravidity, parity, gestational weight gain (GWG) and pre-pregnancy BMI. *p < 0.05.

### 3.4 Association between HbA1c and adverse outcomes in different pre-pregnancy BMI groups

Pre-pregnant normal-weight women with HbA1c ≥5.5% had significantly increased risk of preterm birth (aOR 2.21, 95%CI 1.29,3.78), macrosomia (aOR2.92,95%CI1.52,5.61), PIH (aOR 2.72,95%CI1.36,5.45) and primary C-section (aOR 1.51,95%CI1.01,2.25) compared to pre-pregnant normal weight women with HbA1c ≤5.0%. Interestingly, pre-pregnant underweight women with HbA1c 5.1 - 5.4% at the time of GDM diagnosis were significantly associated with a higher risk of primary C-section compared to their counterparts with HbA1c ≤5.0% (aOR 2.58,1.26,5.26. (**Table 4**).

**Table 4:** Association between HbA1c and adverse outcomes in different pre-pregnancy BMI groups.

	HbA1c% (mmol/mol)		
Pre-pregnancy BMI	≤5.0 (31mmol/mol) (n=755)	5.1≤HbA1c≤ 5.4 (32–36mmol/mol) (n=942)	≥5.5 (37mmol/mol) (n=351)
	aOR (95% CI)	aOR (95% CI)	aOR (95% CI)
Normal (n=1371)			
Preterm birth	Ref	1.31 (0.85,2.01)	2.21 (1.29,3.78)*
Macrosomia	Ref	1.26 (0.72,2.20)	2.92 (1.52,5.61)*
PIH	Ref	1.87 (1.07,3.26)*	2.72 (1.36,5.45)*
Primary C-section	Ref	1.00 (0.75,1.33)	1.51 (1.01,2.25)*
Underweight (n=250)			
Preterm birth	Ref	1.03 (0.33,3.22)	-
Macrosomia	Ref	1.55 (0.22,10.72)	-
PIH	Ref	-	-
Primary C-section		2.58 (1.26,5.26)*	1.24 (0.27,5.60)
Overweight and Obese (n=427)			
Preterm birth	Ref	1.87 (0.75,4.66)	1.34 (0.51,3.52)
Macrosomia	Ref	0.80 (0.33,1.94)	1.75 (0.75,4.07)
PIH	Ref	1.69 (0.78,3.66)	2.12 (0.97,4.62)
Primary C-section	Ref	1.65 (0.88,3.07)	1.51 (0.80,2.86)

**^1^** Glycated hemoglobin A1c, HbA1c; adjusted odds ratio, aOR; confidence interval, CI; pregnancy induced hypertension; reference, Ref.

**^2^** Multiple logistic regression model was adopted and adjusted for gravidity, parity, gestational weight gain (GWG) and maternal age. *p < 0.05.

### 3.5 Association between HbA1c and adverse outcomes in different GWG groups

Interestingly, women with adequate GWG with HbA1c ≥5.5% at the time of GDM diagnosis were significantly associated with risk of PIH (aOR 3.42,95%CI1.48,7.88) compared to their counterparts with HbA1c ≤5.0%. On the other hand, women with inadequate GWG or excess GWG with HbA1c ≥5.5% also showed an increased risk of macrosomia compared to women with inadequate GWG or excess GWG who had HbA1c ≤5.0% (aOR 4.71, 95%CI 1.52,14.58; aOR 3.27,95%CI 1.39,7.71) (**Table 5**).

**Table 5:** Association between HbA1c and adverse outcomes in different GWG groups.

	HbA1c% (mmol/mol)		
GWG	≤5.0 (31mmol/mol) (n=755)	5.1≤HbA1c≤ 5.4 (32–36mmol/mol) (n=942)	≥5.5 (37mmol/mol) (n=351)
	aOR (95% CI)	aOR (95% CI)	aOR (95% CI)
Adequate (n=933)			
Preterm birth	Ref	1.81 (0.96,3.41)	1.42 (0.59,3.38)
Macrosomia	Ref	0.84 (0.44,1.60)	1.59 (0.72,3.51)
PIH	Ref	2.33 (1.11,4.86)*	3.42 (1.48,7.88)*
C-section	Ref	1.38 (0.95,1.99)	1.13 (0.66,1.92)
Inadequate (n=752)			
Preterm birth	Ref	1.19 (0.73,1.95)	1.70 (0.92,3.14)
Macrosomia	Ref	2.44 (0.85,7.00)	4.71 (1.52,14.58)*
PIH	Ref	1.84 (0.86,3.92)	2.27 (0.91,5.69)
Primary C-section	Ref	1.06 (0.72,1.55)	1.59 (0.96,2.63)
Excess (n=363)			
Preterm birth	Ref	1.18 (0.45,3.08)	1.64 (0.58,4.67)
Macrosomia	Ref	1.51 (0.66,3.43)	3.27 (1.39,7.71)*
PIH	Ref	1.64 (0.74,3.62)	2.28 (0.97,5.37)
Primary C-section	Ref	1.22 (0.71,2.12)	1.76 (0.93,3.33)

**^1^** Glycated hemoglobin A1c, HbA1c; adjusted odds ratio, aOR; confidence interval, CI; pregnancy induced hypertension, PIH; reference, Ref.

**^2^** Multiple logistic regression model was adopted and adjusted for gravidity, parity, maternal age and pre-pregnancy BMI. *p < 0.05.

## 4 Discussion

This retrospective study demonstrated a strong relationship between HbA1c at the time of GDM diagnosis (24–28 weeks) and adverse pregnancy outcomes (preterm birth, macrosomia, PIH, and primary C-section) in Chinese women with GDM. Chinese women below recommended HbA1c (6.0%) by ADA might be at high risk of adverse outcomes. In our study, women with HbA1c ≥5.5% had a higher rate of adverse outcomes compared to women with HbA1c 5.1%-5.4% and ≤5.0%. Compared to HbA1c ≤5.0%, HbA1c ≥ 5.5% was significantly associated with an increased risk of macrosomia, preterm birth, PIH, and primary C-section. Our results support the existing evidence that HbA1c might be a biomarker for predicting adverse pregnancy outcomes in GDM women; however, we innovatively demostrated that maternal age, pre-pregnancy BMI, and GWG should be considered when determining the relationship between HbA1c and adverse outcomes. Therefore, our findings may help initiate focused individual prenatal care, health education, and strict counselling to prevent adverse outcomes in high-risk GDM women.

HbA1c during mid-pregnancy have been reported to have the risk of adverse outcomes; however, findings are still controversial. This is due to the measurement of HbA1c in different gestational age, different population involved in the study, and different GDM diagnostic criteria. Given this background, there is still lack of optimum HbA1c for identifying adverse outcomes for GDM women. Surprisingly, HbA1c <5.0% (31mmol/mol) in Asian Indian women with GDM was associated with an increased risk of adverse outcomes (20). A study conducted in Taiwan that included 1989 GDM high-risk women reported that women with mid-pregnancy HbA1c levels lower than 4.5% (26mmol/mol) and higher or equal to 6% (42mmol/mol) were both at increased risk of gestational hypertension, preterm birth, admission to the neonatal intensive care unit, low birth weight, and macrosomia compared to women with HbA1c 4.5%–4.9% (26mmol/mol–30mmol/mol) (21). A study showed that Chinese women above the HbA1c cutoff of 6.0% (42mmol/mol) recommended by the American Diabetes Association (ADA) at the time of GDM diagnosis were at increased risk of primary cesarean section, high birth weight, hypertension during pregnancy, placenta abruption, macrosomia, and neonatal asphyxia compared to women with HbA1c<6.0%(42mmol/mol) (22). In our study, we found that women with HbA1c ≥5.5%might be at increased risk of adverse outcomes, similar to previous studies (17, 23, 24). Zhang Q et al. divided women into two groups including below and above recommended HbA1c cutoff by ADA; however, the sample size of women with HbA1c ≥6.0%(42mmol/mol) was relatively small (49 women), and the risk of adverse outcomes in women with HbA1c<6.0%(42mmol/mol) was not evaluated (22). Therefore, this may explain the differences in our findings. The present study evaluated the association between HbA1c at the time of GDM diagnosis with adverse outcomes in the Asian Chinese population, regardless of recommended HbA1c cutoff <6.0%(42mmol/mol) by ADA. It has been suggested that HbA1c <6.0%(42mmol/mol) cutoff might be higher for Asian women with GDM, thus predisposing them to a higher risk of adverse outcomes (25). It is imperative to note that studies on the association between HbA1c at the time of GDM diagnosis and adverse outcomes were conducted within the Caucasian population, and there is a lack of evidence for the Asian population (17). Therefore, further studies are needed to evaluate the role of HbA1c at the time of GDM diagnosis and determine optimum cutoff of HbA1c for adverse outcomes in Asian women, particularly Chinese women.

Studies have indicated a strong relationship between HbA1c lower than recommended cutoff <6.0%(42mmol/mol) and macrosomia in Asian women with GDM, similar to our findings (20, 21, 25). Although the mechanism is still unknown, according to Hughes et al., relatively higher HbA1c within the normal range at 24 -28 weeks is associated with adverse pregnancy outcomes due to poor glycemic control in the past 12 weeks before GDM diagnosis (26). Additionally, both high HbA1c and excess GWG have been strongly related to the risk of macrosomia offspring in accordance with our findings (27, 28). Pregnant women with excessive GWG have higher levels of amino acids, free fatty acids, and glucose, thus, increasing the risk of high birth weight (29). On the other hand, hyperglycemia leads to macrosomia by glucose crossing the placenta, increasing the utilization of glucose by the fetus and thus increasing fetal adipose tissue (30). Zhang, Q et al. found there’s no significant difference of adverse outcomes in women with inadequate GWG between those with HbA1c ≥6.0%(42mmol/mol) and HbA1c<6.0%(42mmol/mol) (22), contrary to our findings. We noted that women with inadequate GWG with HbA1c levels ≥5.5% (37mmol/mol) had an increased risk of macrosomia compared to women with inadequate GWG women who had HbA1c ≤ 5.0%(31mmol/mol) in accordance with the previous study (31). In the present research, higher HbA1c levels (≥5.5%,37mmol/mol) may contribute to macrosomia in women with insufficient GWG, while a combination of high HbA1c levels and excess GWG might contribute to macrosomia in women with excess GWG. Therefore, strict counselling on lowering HbA1c in women with inadequate GWG and excess GWG might help prevent macrosomia in Chinese women with GDM.

Preterm birth is the leading cause of neonatal mortality and morbidity (32). Contrary to our findings, studies have shown no association between HbA1c and preterm birth (23). We noted that pre-pregnant normal-weight women with HbA1c ≥5.5% (37mmol/mol) and those aged ≥35 years had a significantly higher risk of preterm birth compared to normal-weight women with HbA1c ≤5.0%. Women with inappropriate weight during pregnancy are at increased risk of delivering preterm offspring and severe neonatal morbidity (33, 34). Although the mechanism between weight and preterm birth is still unclear, malnutrition during pregnancy may lead to a lack of essential nutrients, increasing the risk of chronic diseases and inflammation, leading to preterm birth (35). Malnutrition is less likely to be the cause of preterm birth in Zhejiang province; thus, we assume that higher HbA1c in women with normal pre-pregnant BMI might be the leading cause of preterm birth. There are many risk factors for preterm birth; our findings imply that higher HbA1c levels below the ADA-recommended HbA1c cutoff were also likely to lead to preterm birth in normal-weight Chinese women with GDM. Therefore, it is essential to consider the impact of HbA1c on preterm birth, particularly in women with HbA1c≥5.5%(37mmol/mol). Lowering HbA1c by strict blood glucose monitoring and appropriate GWG can help prevent preterm birth, particularly in normal-weight women. However, research may be required to evaluate the relationship between HbA1c and preterm birth, considering all relevant preterm birth-related factors. Solid conclusions on the relationship between HbA1c and preterm birth may help women with GDM prevent preterm birth.

Asian women have lower HbA1c levels compared to other women; thus, the ADA HbA1c cutoff of <6.0%(42mmol/mol) used based on studies that involved only Caucasian women might be higher for Chinese GDM women. An increase in HbA1c is related to the occurrence of microvascular disease, which may play a certain role in the pathogenesis of PIH (36). Moreover, hyperglycemia promotes increased insulin production leading to vascular stenosis, increased vascular resistance, and high blood pressure. Hyperinsulinemia can stimulate the sympathetic nerve, strengthen its excitability, and thus lead to high blood pressure. In the present study, HbA1c was significantly associated with the risk of PIH in women with HbA1c 5.1%-5.4% (32mmol/mol-36mmol/mol) and HbA1c ≥5.5% (37mmol/mol), particularly among women with adequate GWG when compared to women with HbA1c ≤5.0%(31mmol/mol). It is still debatable whether GWG using IOM guidelines is suitable for Chinese GDM women. However, studies show that IOM guidelines may not be appropriate for Chinese women based on the fact that the GWG cutoff by IOM guidelines is based on Caucasian women’s characteristics (37), which might not be suitable for Chinese women. Multiple studies found that GDM women who acquired too much weight during pregnancy had a higher risk of PIH, whereas minimal gestational weight gain was related to a lower risk of hypertensive diseases (14). The possible mechanism is that fat accumulation leads to high estrogen in the body, thus mediating aldosterone secretion, sodium retention caused by the renin-angiotensin system, or directly increasing the recollection of the renal tubules, resulting in hypertension. Another mechanism might be that increased fat accumulation leads to abnormal blood lipid metabolism, which may lead to hypertension. Therefore, using GWG cutoffs based on Chinese women’s characteristics may help Chinese women gain appropriate weight. It is also imperative to note that GWG cutoffs specifically for women with GDM are still lacking. Therefore, more studies on GWG cutoffs in Chinese pregnant women with GDM are warranted. It is imperative to note that gestational weight has been reported as a predictor of glycemic control and adverse pregnancy outcomes in women with GDM (38). Thus, strict GWG monitoring and lowering HbA1c levels may help reduce the risk of PIH in Chinese women with GDM, particularly those with HbA1c 5.1%-5.4% (32mmol/mol- 36mmol/mol) and HbA1c ≥5.5% (37mmol/mol).

In the present study, the association between HbA1c and the risk of primary C-section varied in different pre-pregnancy BMI groups and maternal age groups. Studies have revealed the utility of HbA1c as a biomarker for predicting C-sections (39). Meanwhile, our results also indicated that normal-weight women with HbA1c levels≥5.5% (37mmol/mol) and underweight women with HbA1c 5.1%-5.4% (32mmol/mol – 36mmol/mol) had an increased risk of primary C-section. Antoniou et al. showed that women with pre-pregnancy BMI ≤ 25 kg/m^2^ and HbA1c ≤5.5% (37mmol/mol) had a lower risk of C-section (31). However, women with ≤ 25 kg/m^2^ and HbA1c ≥5.5%(37mmol/mol) were not evaluated in Antoniou et al.’s study. Our findings are in accordance with the HAPO study that showed HbA1c ≥5.8% (at 24 -32 gestational weeks) was significantly associated with an increased risk of primary C- section compared to lower HbA1c levels in pregnant women with hyperglycemia (10). On the other hand, HbA1c in the early trimester at a mean gestational week of 9.25 was significantly associated with primary C-section in non-diabetic Indian women (40). Researchers hypothesize that abnormal glycemia in early pregnancy, which may be indicated by comparatively high HbA1c at the time of GDM diagnosis, is the mechanism underlying the relationship between primary C-section and higher mid-pregnancy HbA1c levels (40). HbA1c reflects glycemia status in the past several weeks; thus, relatively high HbA1c at the time of GDM diagnosis might be associated with poor glycemic control during early pregnancy. It is also important to note that HbA1c at GDM diagnosis that is quite high but still falls within the normal range indicates poor glucose control and is associated with higher odds of adverse outcomes (24, 25); thus, women with relatively high HbA1c within the normal range should not be ignored instead they should be strictly monitored. HbA1c is an independent risk factor of primary C- section (41); however, optimum HbA1c and optimum gestational age at which HbA1c might predict primary C-section remain unknown. While HbA1c at term might provide clinical care information for women at high risk of labor induction or a failed induction (41), HbA1c at term does not offer information on earlier primary and preventive care for women at high risk of adverse outcomes. Our findings on the association between HbA1c at 24 -28 weeks with the risk of primary C-section might have an advantage over findings of HbA1c at term and primary C-section (41), as our findings provided information that can lead to preventive care for GDM women at high risk of primary C-section earlier on, in pregnancy. Studies showed that women who receive strict counselling and follow-up during pregnancy have better glycemic control, a lowered HbA1c level, improved health, and better pregnancy outcomes (42, 43). Therefore, we recommend strict counselling and close follow-up for women with HbA1c 5.1% -5.4%(32mmol/mol-36mmol/mol) and ≥5.5%(37mmol/mol) at 24-28 weeks, particularly those with pre-pregnancy normal weight and underweight BMI for prevention of primary C-section.

While prevention care for pregnant women with diabetes with HbA1c ≥ 6.0%(42mmol/mol) is well established, there is still a lack of specific guidelines on HbA1c to prevent adverse outcomes in GDM. Our findings indicated that even though the recommended HbA1c cutoff for pregnant women with diabetes is <6.0%(42mmol/mol), it is still crucial to consider HbA1c cutoffs specific for women with GDM in consideration of race. Disregarding relatively higher HbA1c within the normal range in Chinese women with GDM can lead to severe adverse pregnancy outcomes (25); thus, earlier counselling and follow-up of women with relatively higher HbA1c(below the recommended ADA HbA1c cutoffs) at the time of GDM diagnosis may reduce the risk of adverse pregnancy outcomes. Nevertheless, further studies are needed to determine an optimum HbA1c cutoff based on Chinese women’s characteristics to prevent adverse outcomes.

To the best of our knowledge, this study is the first to explore the association between HbA1c levels and adverse outcomes considering maternal age, pre-pregnancy BMI, and GWG. Our findings may help healthcare providers to manage GDM pregnant women personally and reduce the risk of adverse outcomes using HbA1c level, pre-pregnancy weight, maternal age, and GWG.

There are several limitations to our study. Firstly, we included a relatively small-size sample. Secondly, there was no further exploration of demographic characteristics, nutrition, and lifestyle, which may influence the results of our study despite the adjustment of confounders. Finally, this was a single-center and retrospective study; further multi-center and future research is required to investigate the utility of HbA1c in predicting adverse outcomes in different ethnicities and gestational age in consideration of pre-pregnant BMI, maternal age, and GWG.

Conclusively, HbA1c is significantly associated with macrosomia, preterm birth, PIH, and primary C-section in GDM women, particularly in women with HbA1c≥5.5%. Our findings may help healthcare providers identify women at high risk of adverse outcomes and manage pregnant women with GDM through counselling and health education by their HbA1c, thereby reducing the incidence of adverse outcomes in GDM. Nonetheless, Chinese women with HbA1c below the recommended HbA1c cut-off are also at high risk of adverse outcomes, which should not be disregarded. Thus, further advanced studies are needed to determine optimal HbA1c cut-offs for predicting adverse outcomes in consideration of Chinese population characteristics. Most importantly, maternal age, pre-pregnancy BMI, and GWG should be considered while evaluating the association between HbA1c and adverse outcomes.

## Data availability statement

The raw data supporting the conclusions of this article will be made available by the authors, without undue reservation.

## Ethics statement

This study was approved by the Human Ethics committee at Women’s Hospital, School of Medicine, Zhejiang University (IRB-20210269-R). The patients/participants provided their written informed consent to participate in this study.

## Author contributions

Conception and design: ZL, DC, and LQ; Analysis and interpretation of the data: All authors; Drafting of the paper: MM and LZ; Paper revision and editing: ZL; Revising paper critically for intellectual content: All authors; Data collection: QW and LZ; Final approval of the version to be published: All authors. All authors agreed to the final content of the manuscript for submission and accountability for all aspects of this work.
